# Psychometric assessment of individual differences in second language reading anxiety for identifying struggling students in classrooms

**DOI:** 10.3389/fpsyg.2022.938719

**Published:** 2022-08-18

**Authors:** Akira Hamada, Shuichi Takaki

**Affiliations:** ^1^Department of English Studies, Kobe City University of Foreign Studies, Kobe, Japan; ^2^Faculty of Human Development and Culture, Fukushima University, Fukushima, Japan

**Keywords:** L2 reading, L2 achievement, individual differences, anxiety, pedagogical screening, a latent rank model

## Abstract

Assessing learners’ individual differences helps identify students who need teacher support in classrooms. Previous studies have examined second language (L2) achievement based on reading anxiety because reading is an input-based activity essential for successful L2 learning. This study applied a latent rank model to identify L2 learners who are likely to be struggling or successful in classrooms according to their L2 reading anxiety symptoms. Moreover, a psychometric function was developed to determine the cutoff anxiety scores that discriminate against their substantial differences. The model was applied to responses from the Foreign Language Reading Anxiety Scale (FLRAS) provided by 335 Japanese learners of English. The results showed that the FLRAS classified students into three ranked groups with ordinal information regarding L2 reading anxiety. Rank 1 exhibited good conditions in L2 reading anxiety. Rank 2 reported high anxiety toward unfamiliar grammar during L2 reading. Rank 3 had even higher anxiety levels, especially for vocabulary and grammatical knowledge deficits and reading difficulty. The cutoff anxiety scores estimated by the model detected students who failed their L2 class with 79% accuracy. Theoretical, methodological, and pedagogical issues in language anxiety were discussed in terms of diagnosis and different approaches to teaching L2 reading.

## Introduction

Second language (L2) anxiety is operationalized as a predictor of the L2 achievement (Teimouri et al., 2019; Zhang, 2019). For example, reading is an input-based activity essential for successful L2 learning but high anxiety toward reading impedes input and intake processing (Horwitz, 2001). L2 reading anxiety is considered influential in the Japanese learners’ achievement in English classrooms (Matsuda and Gobel, 2004) because a task type required for them is mediating a text (e.g., translating and summarizing English documents in Japanese). Mediation activities are in high need in monolingual classrooms and workplaces (Lambert, 2010). Considering that the individual differences in L2 reading anxiety are associated with learning behaviors in a classroom and subsequent L2 achievement (e.g., Sellers, 2000; Alderson et al., 2016; Hamada and Takaki, 2021a, 2021b), it is important to diagnose strengths and weaknesses, identify specific difficulties, and place students into different learning environments.

Ganschow and Sparks (2001) highlighted the importance of pedagogical screening, namely, identifying individuals who are likely to be struggling in L2 classrooms in order to place them in an appropriate learning environment. For example, the Foreign Language Reading Anxiety Scale (FLRAS) developed by Saito et al. (1999) can examine individual differences in anxiety toward L2 reading and identify specific factors evoking L2 reading anxiety (Zhao et al., 2013). Students may further be classified into several groups by predetermined cutoff points (e.g., low, average, and high anxiety groups). While this sort of categorization is practical to determine what groups need a special intervention, some studies showed insignificant associations between L2 achievement and the groups divided by anxiety scores (Phillips, 1992; Marcos-Llinás and Garau, 2009; Wu, 2011). This suggests that the arbitrary cutoff points will cause the misclassification of students.

This study applied a latent rank model to categorize students into ranked groups according to L2 reading anxiety symptoms. The latent rank model is a statistical method that categorizes students into ranked groups (Shojima, 2007). The ranked groups will provide information about what kind of L2 reading anxiety characteristics they have and whether they are struggling learners in L2 classrooms or not. Here, the traditional methods of group categorization are reviewed in terms of L2 anxiety scores and predictive relations to L2 achievement. We then explain the framework and advantages of applying the latent rank model in pedagogical screening. Based on the results of this study, the applicability of the latent rank model and theoretical and pedagogical implications are discussed.

## Literature review

### L2 reading anxiety and achievement

The definition of L2 anxiety is “the worry and negative emotional reaction aroused when learning or using a second language” (MacIntyre, 1999, p. 24). L2 anxiety has been examined using Foreign Language Classroom Anxiety Scale (FLCAS) of Horwitz et al. (1986) based on the idea that anxiety involves a trait, state, and situation-specific construct (MacIntyre and Gardner, 1991; see also Dörnyei and Ryan, 2015). More recently, language-skill-specific anxieties have been examined in terms of their separability: listening, reading, speaking, and writing (Cheng et al., 1999; Saito et al., 1999; Elkhafaifi, 2005; Pae, 2013; Cheng, 2017). In L2 reading, Saito et al. (1999) argued that L2 reading anxiety occurs consistently when performing L2 reading. They developed the FLRAS to reflect the gradation of L2 reading anxiety as a continuous variable and showed that it can be separated from the general L2 anxiety measured by the FLCAS. Each statement of the FLRAS involves two descriptions about a specific situation in L2 reading (e.g., “Whenever I encounter unfamiliar grammar when reading a foreign language”) and a subsequent symptom (e.g., “I get upset”). This psychometric instrument has been adopted to describe individual differences in L2 reading anxiety and investigate the reciprocal relationships between L2 reading anxiety and achievement (e.g., Zhao et al., 2013; Jee, 2016; Sparks et al., 2018a,b; Hamada and Takaki, 2021a) similar to other studies that used the FLCAS (e.g., Horwitz et al., 1986; Phillips, 1992; Ganschow and Sparks, 1996; Hewitt and Stephenson, 2012; Shao et al., 2013).

Comprehensive narrative reviews (MacIntyre and Gardner, 1991; Horwitz, 2001; MacIntyre, 2017) and systematic research syntheses (Teimouri et al., 2019; Zhang, 2019) support the negative relationships between L2 anxiety and achievement including the domain of L2 reading. According to MacIntyre (2017) and MacIntyre and Gardner (1991), the advent of situation-specific approaches to L2 anxiety made a significant contribution to investigating its negative impact on L2 achievement. They indicated initial studies on L2 anxiety produced conflicting findings due to a lack of theoretical (i.e., distinction of state-, trait-, and situation-specific constructs of anxiety) and methodological (i.e., deficits in measurement tools for each anxiety type) sophistications. Horwitz (2001) concluded the negative relationships between L2 anxiety and L2 achievement. Recently, the precise association between L2 reading anxiety and achievement was calculated by two meta-analyses; Teimouri et al. (2019) and Zhang (2019) showed small-to-medium negative correlations of −0.38 (*k* = 8, 95% CI [−0.47, −0.29]) and of −0.23 (*k* = 7, 95% CI [−0.34, −0.11]), respectively.

Although the FLRAS has been validated with respect to the negative relations between L2 reading anxiety and outcome measures, causal inferences based solely on such negative associations have also been criticized. Sparks and his colleagues claimed that the FLRAS merely reflects learners’ self-assessments of their language learning skills when considering several confounding variables affecting both L2 reading anxiety and L2 achievement. For example, FLRAS scores were found to be negatively correlated with first language literacy and literacy-related measures prior to beginning L2 learning (Sparks et al., 2018a). Sparks et al. (2018b) further suggested a mediation model of L2 reading anxiety to raise awareness of spurious correlations with outcome measures. In fact, a mediation analysis by Hamada and Takaki (2021b) indicated that the proportion of variance explained by L2 reading anxiety for achievement significantly decreased when L2 reading proficiency played a mediating role. Several longitudinal studies also demonstrated that the earlier L2 achievement predicted the later development of anxiety (Alamer and Lee, 2021; Sparks and Alamer, 2022).

Despite the limitations to the findings of the negative correlation, L2 reading anxiety has been used to examine L2 achievement (e.g., Wu, 2011; Zhao et al., 2013; Xiao and Wong, 2014; Jee, 2016). However, the continuous scores of the FLRAS are not always informative when identifying students who will be struggling in L2 classrooms due to a lack of information about cutoff points. In such pedagogical screening, a psychometric function has to be applied to the psychometrics to determine the cutoff points that can discriminate the substantial differences of learners’ individual differences (Hasselblad and Hedges, 1995; Finch and French, 2018). This idea is incorporated into testing research as the diagnostic classification models related to the item response theory and diagnostic assessments (Liu and Jiang, 2018, 2020; Ravand and Baghaei, 2020). A review of Ravand and Baghaei (2020) suggested that the diagnostic classification models can compute a psychometric function to classify respondents according to multiple categorical attributes with mastery and non-mastery statuses. Liu and Jiang (2018, 2020) and Shojima (2007, 2008) further developed a graded classification method to discriminate respondents’ latent trait levels.

Establishing cutoff points and psychometric functions could also solve the standard error of measurement with psychometrics problem. Psychological instruments cannot assess the underlying construct without any measurement errors. Therefore, great care should be taken when identifying individual differences in L2 reading anxiety among learners using one-point increments.^1^ Instead, it is pedagogically significant to classify learners into several groups that have substantially different levels of L2 reading anxiety. Converting a continuous variable into categorical groups can inform us if different groups show different L2 reading anxiety symptoms. Such classifications could determine teaching approaches appropriate for particular groups in a classroom (e.g., Ganschow and Sparks, 1991, 2001; Oxford and Ehrman, 1992; Swanson, 2017; Finch and French, 2018; Crowther et al., 2021).

### Establishing cutoff points and the latent rank model

As the *Standards for Educational and Psychological Testing* (American Educational Research Association, 2014) stated, cutoff points must be set on the basis of a clearly defined rationale, including any description of how they are determined. When cutoff points do not function as intended, some students might be misclassified into a group that does not represent their symptoms toward L2 reading anxiety. According to Hasselblad and Hedges (1995), determining cutoff points from continuous scales is known as a discriminant problem, in which cutoff points can be established if the distance between two groups is the largest. This distance is represented by standardized mean differences (i.e., effect sizes) like Cohen’s *d* and Hedge’s *g*. Their meta-analysis also suggested the importance of reporting the exact accuracy of screening tests to reduce misclassification.

However, previous studies have never applied these screening test features to classify students into categorical groups. In case of the FLCAS (Horwitz et al., 1986), Ganschow and Sparks (1996), and Marcos-Llinás and Garau (2009) adopted the method of overall means and standard deviations (*SD*s) in classifications. Students who scored one or more *SD*s above the overall means were identified as a high-anxiety group, those between ±1 *SD*s from the mean were identified as an average-anxiety group, and those with one or more *SD*s below the mean were identified as a low-anxiety group. A similar way to convert anxiety scores is using 25, 50, and 75% quantiles (Phillips, 1992; Hewitt and Stephenson, 2012). Another method used by Shao et al. (2013) determined the definite thresholds like “[s]cores above 132 signify high anxiety; scores between 99 and 132 denote a middle level of anxiety, and scores below 99 imply little or no anxiety” (p. 920).^2^ As Ravand and Baghaei (2020) suggested, their generalizability to other populations cannot be ensured because responses to each questionnaire item depend on both item and respondent traits. Nevertheless, the same classification approach has been adopted in L2 reading anxiety research. Among previous studies included in the meta-analysis by Teimouri et al. (2019), overall means and *SD*s (Wu, 2011), quantiles (Sellers, 2000), and definite cutoff points (Zhao et al., 2013; Xiao and Wong, 2014; Jee, 2016) were employed.

Although L2 anxiety research postulated that students with higher anxiety are more likely to have lower L2 achievement (e.g., Horwitz, 2001), sometimes null or contradicted results were obtained when using the cutoff points set by each study. For example, Sellers (2000) and Wu (2011) showed insignificant differences in L2 reading achievement between low, average, and high anxiety groups. The definite cutoff points were only used to interpret the qualitative differences among student groups (Zhao et al., 2013; Xiao and Wong, 2014; Jee, 2016). By integrating the interview data with the FLRAS scores, Zhao et al. (2013) noted that the items whose average scores were above 3.00 should represent significant sources of L2 reading anxiety. However, these previous studies did not validate whether the cutoff points function as intended by examining the relationships to L2 achievement. These methodological deficits must be resolved to advance theoretical and practical discussions on the relationships between L2 reading anxiety and achievement.

Regarding statistical classification methods, cluster analysis has frequently been used in L2 research on individual differences (Crowther et al., 2021). This technique can identify a number of groups that are different from each other in terms of whether those within a group have similar target characteristics. However, since comparisons across clusters are based on descriptive (e.g., means) and inferential (e.g., analysis of variance) statistics, the cutoff points that differentiate each group will be difficult to reproduce (Pastor et al., 2007). Therefore, recent studies have employed a latent trait approach, such as latent class/profile analysis, to label learners’ individual differences (e.g., Swanson, 2017). In the present study, extended model of the latent profile analysis—the latent rank model—is applied to the FLRAS for screening practicality. Similar to the diagnostic classification models (Liu and Jiang, 2018, 2020), the latent rank model can estimate the number of latent ranks of psychometrics (see Shojima, 2007, 2008, for mathematical details). Similar to latent class/profile analysis, the latent rank model allows for applying the FLRAS’ possible cutoff points to different populations because it incorporates the item response theory to estimate the latent trait of ranked groups. More importantly, latent rank analysis differs from the other methods in that it can identify groups with ordinal information without having to perform *post hoc* comparisons (Shojima, 2009).

In this study, we investigated the number of latent ranks included in the FLRAS that may underlie the diagnostic classification of struggling learners in L2 classrooms. Previous studies using conventional classification methods provide limited perspectives on the characteristics of learners’ individual differences in L2 reading anxiety. The present study attempts to qualitatively categorize the diagnostic information regarding L2 reading anxiety. To that end, the study sought to answer the three research questions below.

1. Are there any cutoff points in the FLRAS for the pedagogical screening of L2 reading anxiety?
2. What kind of L2 reading anxiety characteristics can be diagnosed for each rank estimated by the FLRAS?
3. Can the latent ranks of the FLRAS identify struggling learners in L2 classrooms?

## Materials and methods

### Participants

Participants for the FLRAS latent rank model examination included 335 Japanese learners of English as a foreign language (EFL) from eight classrooms of three universities located in urban, suburban, and rural areas (female = 134, male = 201). Their ages ranged from 18 to 22 years (average = 18.98), and they were taught English as a compulsory school subject from grades 7 to 12. They majored in diverse academic fields, such as the humanities, art, law, social sciences, English, education, engineering, mathematics, chemistry, and business. All participants enrolled in 2–4 English courses for general purposes as required for graduation. Response data from this sample were used to construct a latent rank model that determines the FLRAS’ possible thresholds.

Responses from another sample were collected as a validation dataset that examined whether differences in ranked groups estimated by the latent rank model predicted success levels in L2 (i.e., EFL) classrooms. Data were included from 158 Japanese EFL learners (female = 22, male = 136) from four classrooms of a university located in an urban city. Their ages ranged from 18 to 19 years (average = 18.32), and they had been taught English as a compulsory school subject from grades 7 to 12. Their major was engineering. At the university, they enrolled in an English course for general purposes during the survey.

### Materials

#### The foreign language reading anxiety scale

A Japanese-translated version of the FLRAS (Hamada and Takaki, 2021a) was used to measure Japanese EFL students’ reading anxiety (see Table 1) because the assessment by this scale was more comprehensive than any of the other brief measurements (Cheng, 2017). The word English in each statement was used instead of the original words French, Russian, and Japanese in the FLRAS (Saito et al., 1999, pp. 205–207). This psychometric instrument consisted of 20 self-report items with a five-point Likert scale: (1) strongly disagree, (2) disagree, (3) neither agree nor disagree, (4) agree, and (5) strongly agree. The sequence of the questionnaire statements was rearranged using a random-number method.

**Table 1:** Means with 95% CIs and SDs for each Foreign Language Reading Anxiety Scale (FLRAS) statement.

No.	Statements	*M*	95% CI	*SD*
Factor 1: Reading difficulty (Cronbach’s α = 0.82, 95% CI [0.78, 0.87])				
1.	I get upset when I am not sure whether I understand what I am reading in English.	3.60	[3.50, 3.70]	0.95
2.	When reading English, I often understand the words but still cannot quite understand what the author saying.	3.28	[3.17, 3.39]	1.01
3.	When I am reading English, I get so confused I cannot remember what I am reading.	3.20	[3.08, 3.31]	1.05
4.	I feel intimidated whenever I see a whole page of English in front of me.	3.27	[3.14, 3.40]	1.18
5.	I am nervous when I am reading a passage in English when I am not familiar with the topic.	2.87	[2.76, 2.99]	1.08
6.	I get upset whenever I encounter unknown grammar when reading English.	3.56	[3.45, 3.67]	0.99
7.	When reading English, I get nervous and confused when I do not understand every word.	3.44	[3.34, 3.55]	0.95
8.	It bothers me to encounter words I cannot pronounce while reading English.	2.61	[2.49, 2.73]	1.11
9.	I usually end up translating word by word when I’m reading English.	2.94	[2.83, 3.06]	1.05
Factor 2: Self-efficacy in reading (Cronbach’s α = 0.77 [0.73, 0.81])				
12.	I enjoy reading English.	2.73	[2.62, 2.84]	1.03
13.	I feel confident when I am reading in English.	2.45	[2.33, 2.56]	1.06
14.	Once you get used to it, reading English is not so difficult.	3.26	[3.15, 3.37]	1.00
15.	The hardest part of learning English is learning to read.	2.76	[2.66, 2.86]	0.92
16.	I would be happy just to learn to speak English rather than having to learn to read as well.	3.35	[3.24, 3.45]	1.00
17.	I do not mind reading to myself, but I feel very uncomfortable when I have to read English aloud.	2.81	[2.69, 2.93]	1.13
18.	I am satisfied with the level of reading ability in English that I have achieved so far.	1.88	[1.78, 1.98]	0.89
Factor 3: Language distance (Cronbach’s α = 0.72 [0.68, 0.76])				
10.	By the time you get past the funny letters and symbols in English, it is hard to remember what you are reading about.	2.81	[2.70, 2.93]	1.06
11.	I am worried about all the new symbols you have to learn in order to read English.	2.75	[2.62, 2.84]	1.09
19.	English culture and ideas seem very foreign to me.	2.17	[2.06, 2.27]	0.98
20.	You have to know so much about English history and culture in order to read English.	3.13	[3.03, 3.24]	1.00

*n* = 335.

Based on the factor structure of the FLRAS (Matsuda and Gobel, 2004; Hamada and Takaki, 2021a; see also Saito et al., 1999), each item was labeled as reading difficulty (Items 1–9), self-efficacy in reading (Items 12–18), and language distance (Items 10–11 and 19–20). As Saito et al. (1999) suggested, these specific statements could be qualitatively interpreted as different situation-specific anxieties that might interfere with L2 learning. Specifically, low anxious students are more likely to be full of self-efficacy in L2 reading and subsequently reach high L2 achievement (Mills et al., 2007). The language distance indicates specific anxieties toward unfamiliar writing systems and cultural material (Saito et al., 1999).

#### L2 reading proficiency test

The standardized English reading proficiency test (TOEIC Bridge®; Educational Testing Service, 2007) was used to measure participants’ L2 reading proficiency. It had a multiple-choice format and consisted of 50 items. Responses were marked dichotomously (score range = 0–50). The test scores were used to examine the association between L2 reading anxiety and proficiency. As defined in language testing (Bachman and Palmer, 2010), the reading proficiency test evaluated a static trait of learners’ reading skills while the L2 achievement reflected mastery of the just-completed courses in which students were enrolled (Ross, 1998; see also Teimouri et al., 2019; Zhang, 2019).

#### L2 course achievement assessment

The course grade from the other sample was used to indicate L2 achievement (see also Zhang, 2019). Since there were no participants with learning disabilities, this study defined struggling students as those who might drop out from a classroom even if they continued to learn to read. As noted, participants took the achievement test in partial fulfillment of their English course for general purposes. The test consisted of integrated reading-to-write task performance (40%), independent listening skills (40%), and spoken interaction (20%) that were introduced and practiced in the L2 classrooms to evaluate the degree to which participants achieved learning goals (Bachman and Palmer, 2010). The rating categories of the university were excellent (90–100), very good (80–89), good (70–79), fair (60–69), and failing (0–59). The course grade was used as a dependent variable to explore whether the psychometric function could predict the participants’ success (i.e., excellent to good) and fair-failing in the classroom.^3^

### Procedure

The survey was conducted during the authors’ regular L2 classes. Participants were notified of the study’s purpose and how their personal data would be used. They provided written informed consent.

First, the L2 reading proficiency test was implemented in 35 min. Next, the participants received detailed information on how to answer the FLRAS and completed 20 self-report items at their own pace. They were also asked not to answer the questions based on the specific class in which the questionnaire was administered (see Matsuda and Gobel, 2004; Hamada and Takaki, 2021a). There was no set time limit but the administration time was approximately 15 min. Apart from the survey, the end-of-quarter test for the L2 achievement assessment of the other sample was conducted approximately 2 months after the FLRAS had been implemented to examine whether the preceding L2 reading anxiety affected the degree of success in the L2 classroom.

### Data analysis

Questionnaires with missing values (0.89%) were excluded resulting in the final sample of 335 participants. The reverse code scale items (Items 12, 13, 14, and 18) were corrected after reporting the descriptive statistics (see Table 1) so that a high value manifests the same type of response on the other items. An item-total correlation analysis showed no negatively correlated items with the total anxiety scores (range = 0.00–0.65). All the materials and data used in this study are available at the IRIS Digital Repository.

To answer the first research question, a self-organized mapping neural network was adapted in a latent rank analysis using Exametrika version 5.5 (Shojima, 2019). Following Shojima (2008), two criteria were considered to determine the number of latent ranks of the FLRAS. First, the estimated ranks were aligned ordinally and the principal components increased monotonically because the observed data contained ordinal graded responses. Under this condition, the latent rank model that fit the observed data best was selected based on the Akaike information criterion (AIC) and Bayesian information criterion (BIC). Then, the probabilities of which ranked group the participants belonged to were calculated (i.e., rank membership profile; Shojima, 2007). The thresholds of L2 reading anxiety scores between the adjacent two ranks were identified when certain anxiety scores significantly changed the rank membership profile. For example, an anxiety score of 60 indicated if a participant belonged in Rank 1 or 2 with a 60 and 40% probability, respectively, and a score of 61 indicated if a participant belonged to Rank 1 or 2 with a 40 and 60% probability, respectively, the cutoff point for discriminating between Rank 1 and 2 was determined as the anxiety score of 61.

In relation to the second research question, an implicational analysis was conducted to describe the L2 reading anxiety characteristics of each ranked group. The implicational analysis and subsequent scaling are methods to display individual and group variations of data to reveal both underlying systematicity in the data and a theoretical explanatory model (Andersen, 1978). In this study, the group average scores for each item were further rounded to the nearest first decimal point to examine which FLARS items participants responded to positively and negatively. Namely, the scores of 1.00–1.49, 1.50–2.49, 2.50–3.49, 3.50–4.49, and 4.50–5.00 were converted to 1, 2, 3, 4, and 5, indicating the participants *strongly disagreed*, *disagreed*, *neither disagreed nor agreed*, *agreed*, and *strongly disagreed* with particular statements. Using this approximated data, an implicational scaling was created, in which the questionnaire items were listed in descending order from the least to most anxious situations in L2 reading as perceived by participants.

Finally, the third research question was investigated by binominal logistic regression to predict the probabilities of participants’ success in L2 classrooms based on their L2 reading anxiety. L2 achievement was an indicator of success in the classroom, binarily converted into “Success” (> = 70: Grades Excellent, Very Good, and Good) and “Fair-Failing” (< 70: Grades Fair and Failing). To evaluate the detective power for pedagogical screening, 70% of the observed data was randomly split into a training set for building a detective model. The remaining data were used as a test set for evaluating this model. In addition, this study compared two mediation models to evaluate the direct effect of L2 reading anxiety even when L2 reading proficiency was a mediating variable. If the L2 reading anxiety merely reflected the learners’ self-perception of L2 reading difficulties, its direct effect on L2 achievement would disappear (i.e., a complete mediation model). In contrast, it could be possible that the direct effect of L2 reading anxiety remained significant while L2 reading proficiency played a mediating role. These analyses were conducted using R-4.1.3 (R Core Team, 2021).

## Results

### The FLRAS cutoff points

Table 1 displays the descriptive statistics of the FLRAS. The measurement reliability was adequate (Cronbach’s α = 0.83, 95% CI [0.81, 0.86]). The descriptive statistics for total FLRAS scores were as follows: *M* = 61.71, 95% CI [60.63, 62.79], *SD* = 10.02, *Min* = 28, *Max* = 91, and *SE* = 0.55. Therefore, the standard error of measurement for the FLRAS was 4.12. The descriptive statistics of the L2 reading proficiency test were as follows: *M* = 31.61, 95% CI [30.55, 32.67], *SD* = 9.89, *Min* = 4, *Max* = 49, and *SE* = 0.54. Internal consistency of the test was adequately high (Cronbach’s α = 0.91, 95% CI [0.89, 0.93]). According to the 95% CIs of the means, no floor or ceiling effects were found.

**Figure 1:**
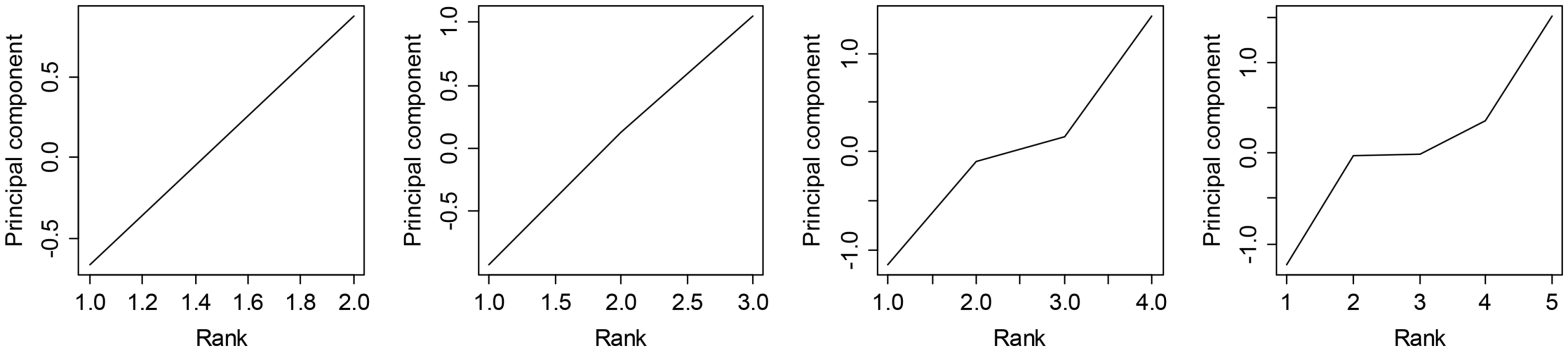
Changes in the principal component values for the 2- to 5-rank models.

Figure 1 shows changes in the principal components from 2- to 5-rank models. This indicated the principal components increased monotonically only in the 2- and 3-rank models. In contrast, the results suggested no substantial differences in L2 reading anxiety between Ranks 2 and 3 in the 4-rank model and between Ranks 2, 3, and 4 in the 5-rank model. The observed data fit the 3-rank model (AIC = 18,215; BIC = 18,680) better than the 2-rank model (AIC = 18,536; BIC = 18,845). Therefore, the subsequent analyses were conducted using the 3-rank model of the FLRAS.

**Figure 2:**
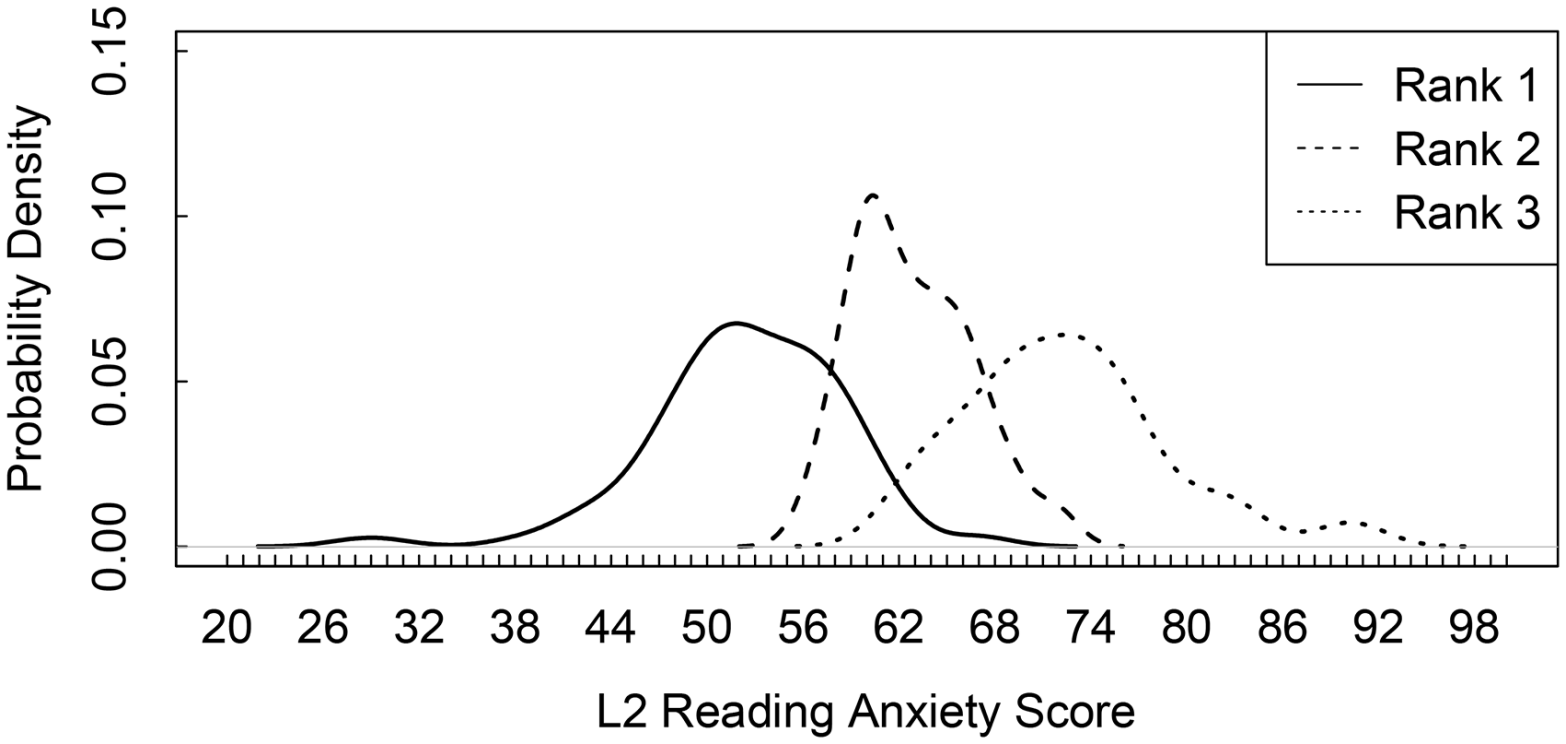
Probability density curves of the rank membership profiles. Two vertical lines indicate the thresholds between the adjacent ranks.

Table 2 displays the descriptive statistics of L2 reading anxiety for each rank and thresholds between the adjacent two ranks. A Kruskal–Wallis test^4^ showed significant differences in the L2 reading anxiety scores between the adjacent two ranks, χ^2^(2) = 257.86, *p* < 0.001, with large effect sizes (Ranks 1–2: *p* < 0.001, *d* = 2.00, 95% CI [1.68, 2.32]; Ranks 2–3: *p* < 0.001, *d* = 1.84, 95% CI [1.51, 2.17]). This suggests that the L2 reading anxiety scores considerably increased as per ranking. The thresholds were the anxiety scores where the probabilities of the participants belonging to each ranked group differed between the adjacent two ranks. As shown in Figure 2, participants with anxiety scores below 57 were highly likely to belong to Rank 1. Participants with anxiety scores between 58 and 67 were grouped into Rank 2. Participants with anxiety scores above 68 were in Rank 3, showing the highest L2 reading anxiety.

**Table 2:** Differences in L2 reading anxiety, its subscales, and L2 reading proficiency between three latent ranks.

	Rank 1 (*n* = 132)			Rank 2 (*n* = 101)		Rank 3 (*n* = 102)			
Measures	*M*	95% CI	*SD*	*M*	95% CI	*SD*	*M*	95% CI	*SD*
Overall L2 reading anxiety	52.50	[51.46, 53.54]	6.05	62.84	[62.11, 63.58]	3.72	72.50	[71.24, 73.76]	6.43
Reading difficulty	2.66	[2.57, 2.75]	0.53	3.32	[3.25, 3.39]	0.36	3.88	[3.78, 3.97]	0.48
Self-efficacy in reading	2.82	[2.75, 2.90]	0.43	2.99	[2.92, 3.07]	0.38	2.66	[2.56, 2.76]	0.51
Language distance	2.08	[1.99, 2.17]	0.52	2.78	[2.67, 2.89]	0.54	3.23	[3.11, 3.35]	0.61
L2 reading proficiency	35.45	[33.96, 36.93]	8.58	29.31	[27.22, 31.40]	10.59	29.57	[27.81, 31.32]	8.93

The thresholds between Ranks 1 and 2 and Ranks 2 and 3 were 57/58 and 67/68, respectively.

### Diagnostic characteristics of L2 reading anxiety

A Kruskal–Wallis test showed a significant main effect of L2 reading anxiety on L2 reading proficiency, χ^2^(2) = 30.98, *p* < 0.001 (see Table 2). The participants in Rank 1, who showed the least L2 reading anxiety, had better L2 reading proficiency than those in Rank 2 (*p* < 0.001, *d* = 0.65, 95% CI [0.38, 0.91]) and in Rank 3 (*p* < 0.001, *d* = 0.67, 95% CI [0.40, 0.94]). In contrast, there was no significant difference between Ranks 2 and 3 in L2 reading proficiency (*p* = 0.842, *d* = −0.03, 95% CI [−0.30, 0.25]).

Table 3 shows changes in average response scales for each item from Ranks 1 to 3. Item discriminability^5^ also indicates how big differences among the three ranks were found. As overall results indicated that the anxieties manifested by each statement were likely to increase from Ranks 1 to 3, the FLRAS could discriminate the individual differences in L2 reading anxiety. Specifically, anxiety toward reading difficulty (Items 1–9) was a strong discriminator of the learners (range = 0.45–0.76). Although self-efficacy in reading also discriminated the characteristics of the three ranks (range = 0.31–0.49), Items 16 (0.12) and 18 (0.23) showed less discriminative power. Language distance was also able to identify differences between the three ranks by Items 10 (0.58) and 11 (0.65), but not by Items 19 (0.25) and 20 (0.15).

**Table 3:** Average response scales for each item among the three ranks and item characteristics.

	Rank 1 (*n* = 132)		Rank 2 (*n* = 101)		Rank 3 (*n* = 102)		Item
Item number and labels	*M*	*SD*	*M*	*SD*	*M*	*SD*	discriminability
1: Reading difficulty	3.14	1.05	3.48	0.63	4.33	0.57	0.52
2: Reading difficulty	2.85	1.01	3.33	0.71	3.79	1.03	0.48
3: Reading difficulty	2.58	0.99	3.27	0.66	3.92	0.94	0.58
4: Reading difficulty	2.52	1.07	3.32	0.86	4.20	0.90	0.76
5: Reading difficulty	2.17	0.83	3.26	0.77	3.40	1.15	0.59
6: Reading difficulty	3.05	1.06	3.50	0.70	4.27	0.69	0.51
7: Reading difficulty	2.94	1.02	3.47	0.64	4.08	0.68	0.47
8: Reading difficulty	2.05	0.93	2.95	0.80	3.00	1.29	0.45
9: Reading difficulty	2.44	0.99	2.85	0.80	3.69	0.92	0.54
10: Language distance	2.20	0.81	2.74	0.77	3.69	0.98	0.58
11: Language distance	2.07	0.89	2.94	0.72	3.43	1.13	0.65
12: Self-efficacy in reading	2.34	0.99	2.80	0.63	3.18	1.20	0.36
13: Self-efficacy in reading	3.10	1.07	3.44	0.75	4.25	0.95	0.49
14: Self-efficacy in reading	2.40	0.88	2.72	0.72	3.20	1.19	0.41
15: Self-efficacy in reading	2.36	0.80	2.95	0.70	3.10	1.07	0.31
16: Self-efficacy in reading	3.31	1.03	3.32	0.79	3.42	1.14	0.12
17: Self-efficacy in reading	2.11	0.96	3.20	0.71	3.32	1.20	0.48
18: Self-efficacy in reading	4.19	0.80	3.55	0.91	4.59	0.67	0.23
19: Language distance	1.61	0.70	2.74	0.81	2.32	1.05	0.25
20: Language distance	3.08	1.13	3.03	0.71	3.31	1.04	0.15

High values of Items 12, 13, 14, and 18 (reverse coded) indicate high anxiety. Generally, the discriminability among ranks became low when the items did not show monotonic increase.

Table 4 shows an implicational scaling that describes the different participant characteristics by the ranked group. Overall, anxiety toward language distance was not a stronger cause of L2 reading anxiety than the other two factors. While the factor of self-efficacy in reading also showed similar results, Item 13 was related to relatively high anxiety on the scale. Statements regarding reading difficulty were located at the relative bottom of the implicational scaling. This suggested that anxiety toward reading difficulty was the major source of L2 reading anxiety. More specifically, participants in Rank 1 responded, “*disagree*” and “*neither disagree nor agree*” for almost all statements. Participants in Rank 2 also neither disagreed nor agreed to the statements but showed high anxiety toward unfamiliar grammatical features during L2 reading (Item 6). Participants in Rank 3 were likely to negatively respond to statements regarding reading difficulty and confidence in L2 reading (Item 13). The orthographic differences between Japanese and English were also a source of their high L2 reading anxiety (Item 10).

**Table 4:** Implicational analysis summary results.

	Approximated response scale					
Item number and labels	Rank 1		Rank 2		Rank 3	
11: Language distance	low	2	average	3	average	3
12: Self-efficacy in reading	low	2	average	3	average	3
14: Self-efficacy in reading	low	2	average	3	average	3
15: Self-efficacy in reading	low	2	average	3	average	3
17: Self-efficacy in reading	low	2	average	3	average	3
5: Reading difficulty	low	2	average	3	average	3
8: Reading difficulty	low	2	average	3	average	3
10: Language distance	low	2	average	3	high	4
3: Reading difficulty	low	2	average	3	high	4
4: Reading difficulty	low	2	average	3	high	4
9: Reading difficulty	low	2	average	3	high	4
1: Reading difficulty	average	3	average	3	high	4
2: Reading difficulty	average	3	average	3	high	4
7: Reading difficulty	average	3	average	3	high	4
13: Self-efficacy in reading	average	3	average	3	high	4
6: Reading difficulty	average	3	high	4	high	4

Items 16, 18, 19, and 20 were removed from the implicational scaling due to extremely low item discriminability.

### Pedagogical screening

The practicality of the FLRAS thresholds was investigated using the other sampled population. Table 5 shows the descriptive statistics of their L2 reading anxiety scores and L2 achievement assessment for the three ranked groups. Kruskal–Wallis tests showed significant main effects of the ranked groups on both L2 reading anxiety, χ^2^(2) = 107.34, *p* < 0.001, and L2 achievement, χ^2^(2) = 34.78, *p* < 0.001. Multiple comparisons with Holm’s adjustment demonstrated that the participants in Rank 3 reached considerably less L2 achievement than Rank 1 (*p* < 0.001, *d* = 1.51, 95% CI [0.98, 2.04]) and Rank 2 (*p* < 0.001, *d* = 1.66, 95% CI [1.17, 2.15]). There was no outstanding difference between Rank 1 and Rank 2 (*p* = 0.650, *d* = 0.09, 95% CI [−0.45, 0.27]), although their L2 reading anxiety scores differed substantially (*p* < 0.001, *d* = 2.86, 95% CI [2.36, 3.36]). The correlation between their L2 reading proficiency and achievement was *r* = 0.37 (95% CI [0.27, 0.46]), suggesting both tests measured different constructs of L2 performance as intended (Ross, 1998).

**Table 5:** Means with 95% CI and SD for L2 reading anxiety and L2 achievement.

		L2 reading anxiety			L2 achievement		
Groups	*n*	*M*	95% CI	*SD*	*M*	95% CI	*SD*
Rank 1	48	53.23	[52.19, 54.27]	3.58	82.79	[79.52, 86.07]	11.28
Rank 2	82	62.33	[61.69, 62.97]	2.93	83.76	[81.36, 86.15]	10.91
Rank 3	28	70.57	[69.56, 71.58]	2.60	64.43	[59.17, 69.69]	13.56

The L2 achievement test reliability was adequate [Cronbach’s α = 0.74, 95% CI (0.68, 0.80)].

**Figure 3:**
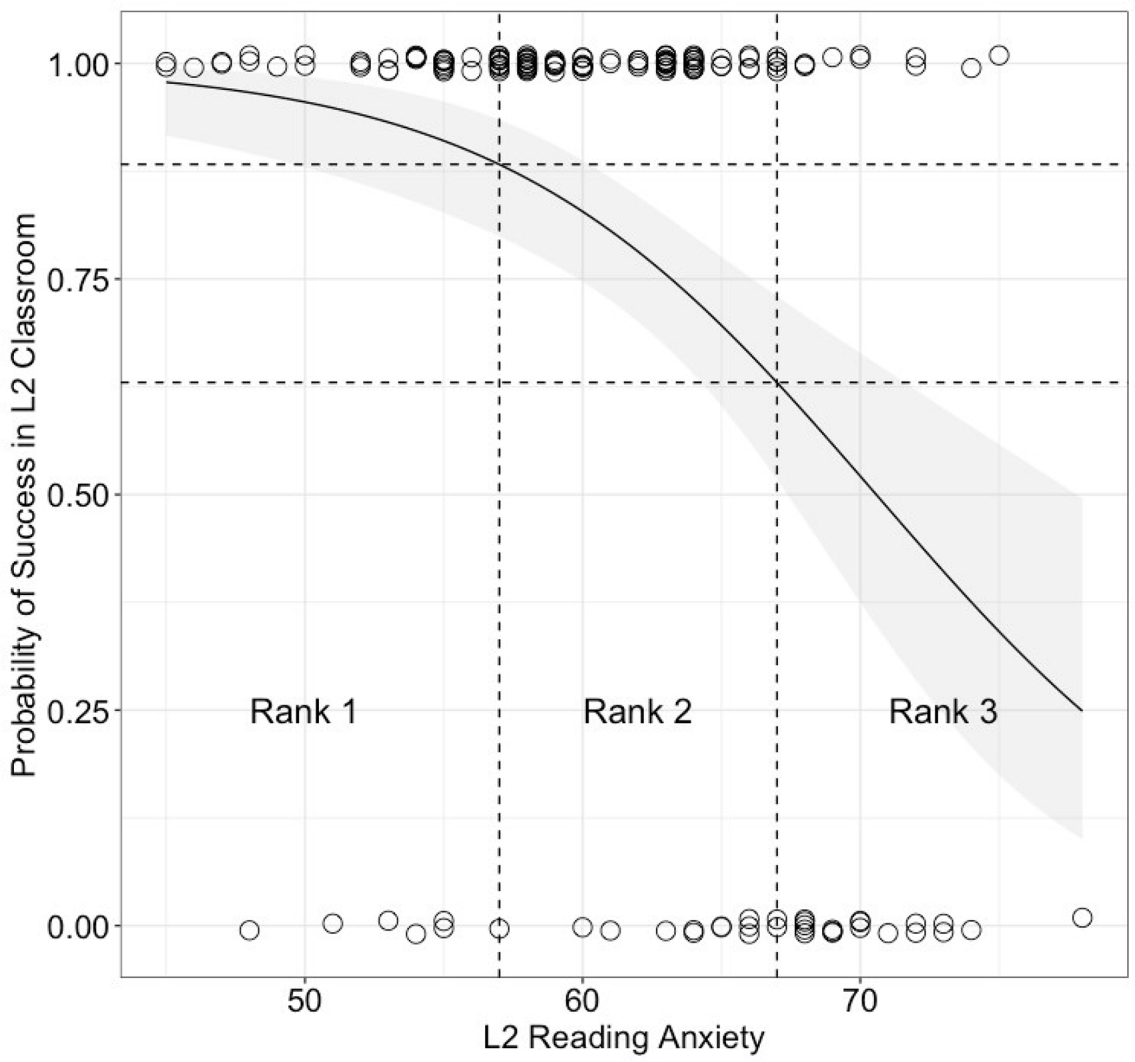
A probability curve with a 95% CI of the success in the L2 classrooms modeled by the logistic regression. A jitter-plot represents the actual points of each observation (The ratio of success to fair-failing: Rank 1 = 41/7, Rank 2 = 70/12, and Rank 3 = 10/18). Dashed lines indicate the thresholds of the L2 reading anxiety scores that discriminate between the probabilities of the success in the L2 classrooms and Ranks 1–3.

A logistic regression model established by the training dataset showed that L2 reading anxiety explained the variances of success probabilities in the L2 classrooms (β = −0.15, *SE* = 0.04, *z* = −4.16, *p* < 0.001). The psychometric function, predicting the outcome of an observation given a predictor variable (L2 reading anxiety), is an S-shaped curve. As plotted in Figure 3, the FLRAS thresholds indicated that the probability of success in L2 classrooms that differentiated between Ranks 1 and 2 was 88%. Such probability between Ranks 2 and 3 was 63%. The accuracy rate for detecting the struggling students in the L2 classrooms was 79% in the test dataset.

**Figure 4:**
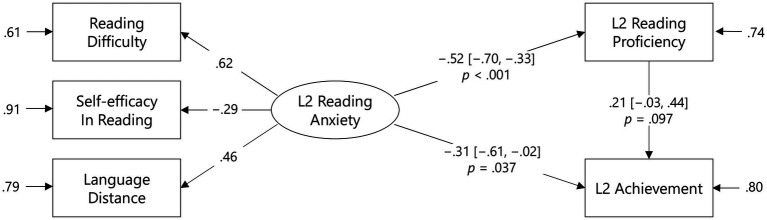
A mediation model of the effects of L2 reading anxiety on L2 achievement. Values in brackets are 95% CIs.

**Figure 5:**
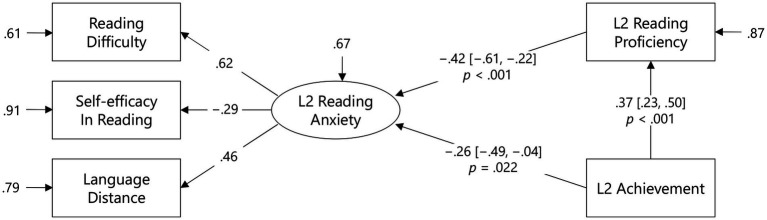
A mediation model of the effects of L2 achievement on L2 reading anxiety. Values in brackets are 95% CIs.

Finally, Figure 4 shows the standardized path coefficients from L2 reading anxiety to proficiency (β = −0.52, 95% CI [−0.70, −0.33], *p* < 0.001), from proficiency to achievement (β = 0.21, 95% CI [−0.03, 0.44], *p* = 0.097), and from anxiety to achievement (β = −0.31, 95% CI [−0.61, −0.02], *p* = 0.037). These results indicate a partial mediation model, in which L2 reading anxiety affected the degree of L2 achievement partially because of the mediating role of L2 reading proficiency. Importantly, Figure 5 indicates that the direct effect of L2 achievement on L2 reading anxiety was also significant (β = −0.26, 95% CI [−0.49, −0.04], *p* = 0.022). This model fit the observed data (AIC = 1,748, BIC = 1,779) better than the former model (AIC = 3,021, BIC = 3,055). Taken together, although the mediating effects of L2 reading proficiency can never be ignored, the direct effect of L2 reading anxiety might be considered for the factor affecting pedagogical screening. However, it is highly possible that the degree of L2 achievement determined the magnitude of L2 reading anxiety.

## Discussion

This study applied a latent rank model to the FLRAS for pedagogical screening of the students who would be struggling in L2 classrooms. Reading is an essential cognitive activity for L2 learning (e.g., Grabe, 2009) but demanding for learners who feel highly anxious toward reading in an L2 (Saito et al., 1999; Sellers, 2000; Matsuda and Gobel, 2004; Zhao et al., 2013; Jee, 2016; Hamada and Takaki, 2021a,b). Because high L2 reading anxiety can be associated with reading attitude in a classroom (Yamashita, 2007), we predicted that particular groups of learners who showed certain symptoms of L2 reading anxiety led to different levels of L2 achievement. The latent rank model provided evidence that the FLRAS can diagnose L2 reading anxiety of struggling students in L2 classrooms. The three discrete groups showed different symptoms of L2 reading anxiety and L2 achievement. Moreover, the psychometric function applicable to the FLRAS could predict the probability of success in L2 classrooms with 79% accuracy. In line with these findings, the theoretical and methodological issues for psychometric assessment of individual differences in L2 reading anxiety will be discussed.

The first research question addressed FLRAS cutoff points that can discriminate differences in L2 reading anxiety among groups of L2 learners. The results showed that it could differentiate the characteristics among only three groups. Differences among ranked groups were not clear for classifying participants into four or more groups (see Figure 1). The FLRAS’ standard error of measurement also indicated that the true score of L2 reading anxiety per participant varied from −4.12 to 4.12. These findings suggested the FLRAS was not reliable enough to discriminate L2 learners on its 20–100 continuous scale. Although previous studies have used the raw scores (e.g., Saito et al., 1999; Matsuda and Gobel, 2004; Wu, 2011; Zhao et al., 2013; Xiao and Wong, 2014; Jee, 2016), it should be noted that individual anxiety scores do not always reflect substantial differences in individual L2 reading anxiety.

Specifically, the latent rank analysis showed the score range of the FLRAS can be mapped into a three-point discrete scale. By grouping participants with the latent rank information, their L2 reading proficiency was found to significantly differ between the low-anxiety group (Rank 1) and the other two groups (Ranks 2 and 3). Consistent with the present result, differences between average- and high-anxiety groups were sometimes unclear in previous studies (Phillips, 1992; Ganschow and Sparks, 1996; Hewitt and Stephenson, 2012). However, these studies commonly provided evidence that the low-anxiety group was always the most proficient in L2 proficiency tests. Although there were differences in the questionnaires used, the present result was consistent with Ganschow and Sparks (1996) showing that the low-anxiety group was the most proficient in L2 reading. Given the relatively weak correlations between L2 anxiety and proficiency (Teimouri et al., 2019; Zhang, 2019), it is reasonable that group differences in L2 proficiency were not large.

The second research question explored what kind of characteristics can be diagnosed for each ranked group by the FLRAS. The results of the implicational analysis found qualitative differences between the three ranked groups (see Table 5). More specifically, reading difficulty was the strongest factor that differentiated the ranked groups, followed by self-efficacy in reading, and language distance. This result was fully consistent with previous studies that showed the relative proportions of variances explained by these three factors. Matsuda and Gobel (2004) and their replication study (Hamada and Takaki, 2021a) revealed that reading difficulty explained the largest variance of the FLRAS responses, followed by self-efficacy in reading and language distance. The result also supported the evidence that cognitive processes and linguistic knowledge are major components of L2 reading (Grabe, 2009), resulting in a source of perceived reading difficulty.

More specifically, participants categorized into Rank 1 exhibited good conditions in L2 reading anxiety. They responded with less impacts for differences in orthographic features and writing system on their L2 reading anxiety (Items 10 and 11). Reading was also a part of their enjoyment (Item 12) and not difficult to learn in L2 classrooms (Items 14, 15, and 17) even though their confidence in L2 reading was slightly high (Item 13) compared to the other specific situations of self-efficacy. Reading difficulty caused by cognitive processing involved in L2 reading did not make them uneasy (Items 3, 4, 5, 8, and 9). Instead, anxieties toward linguistic knowledge such as unfamiliar words (Item 7) or grammar (Item 6) were higher among participants in Rank 1.

Participants in Rank 2 showed similar trends, only responding negatively to unfamiliar grammar during L2 reading. However, their anxieties toward several aspects substantially increased compared to participants in Rank 1. First, the level of L2 reading anxieties related to language distance (Items 10 and 11) and self-efficacy (Items 12, 14, 15, and 17) increased from low to average. Likewise, perceived reading difficulty of participants in Rank 2 was generally higher than that of Rank 1. The L2 reading anxiety of Rank 3 spiked even further, particularly regarding several reading difficulties. Their anxiety levels were on average only toward unfamiliar topics of a passage (Item 5) and word decoding (Item 8) in L2 reading. Compared to participants in Ranks 1 and 2, they did not feel confident during L2 reading. The orthographic differences between Japanese and English were also a source of their high L2 reading anxiety (Item 10). In contrast, their self-efficacy in L2 reading did not differ from Rank 2 students. These results suggest that while highly anxious students perceived their L2 reading ability as low due to insufficient cognitive processing, they might feel that L2 reading is not fun, but not painful either.

These qualitative differences among the ranked groups highlight the importance of considering the relative influences of situation-specific reading anxiety when interpreting the FLRAS responses. Previous studies provided diagnostic information by comparing different cultural groups of learners (Saito et al., 1999) and qualitative analyses of interview protocols (Zhao et al., 2013). Other studies used definite cutoff points based on the Likert-scale (Xiao and Wong, 2014; Jee, 2016). The present findings added a more fine-grained view that the FLRAS can diagnose individual differences in L2 reading anxiety. Such diagnostic information is useful to identify the strengths and weakness of L2 readers (Alderson et al., 2016) and examine relationships with L2 learning problems that lead to L2 achievement (Ganschow and Sparks, 1991, 2001).

Finally, the third question was related to the practical, but ignored use of the FLRAS and other psychometrics in L2 anxiety research. The results showed the psychometric function of the FLRAS could accurately identify students who were likely to be successful or struggling in L2 classrooms. In other words, L2 reading anxiety played a significant role in the odds of being successful L2 learners or not (Alderson et al., 2016). No doubt, variations related to high and low perceptions of L2 reading anxiety helped guess who would be struggling in L2 classrooms and those considered good L2 readers, respectively. In fact, the probabilities of success in L2 classrooms varied considerably according to the three ranked groups. As shown in Figure 3, the S-shaped curve for Rank 1 was a gradual slope compared to Ranks 2 and 3. This suggested that a student labeled as a prospectively successful L2 learner (Rank 1) was likely to achieve particular learning goals in L2 classrooms. The aforementioned results supported this finding because Rank 1 students were likely to manifest the lowest anxiety toward reading difficulty and language distance. They were also full of self-efficacy despite relatively low confidence in L2 reading. These arguments were consistent with several studies that showed individual differences in L2 reading anxiety as the psychological factors defining strengths of successful L2 readers (Saito et al., 1999; Mills et al., 2007; Zhao et al., 2013; Xiao and Wong, 2014; Alderson et al., 2016; Jee, 2016).

Figure 3 also shows many Rank 2 students were successful in their classrooms. Because they did not show high L2 reading anxiety with respect to reading difficulty, self-efficacy in reading, and language distance, the means of their L2 achievement test did not differ from those of students in Rank 1. However, the actual data points indicated the growth of the number of students who received a fair or failing grade around the threshold between Ranks 2 and 3. In line with this result, the probability of success in L2 classrooms dropped to 63% as the students’ L2 reading anxiety score approached to 67. Although the implicational analysis did not produce any characteristics of the L2 reading anxiety of Rank 2, it should be interpreted with caution when they showed relatively strong overall L2 reading anxiety. Particularly, students who manifested strong anxiety toward unfamiliar grammar and much less confidence in L2 reading could be labeled as potentially unsuccessful in L2 classrooms (see also Zhao et al., 2013).

As noted, students in Rank 2 were not found to be prospectively unsuccessful in L2 classrooms, although their L2 reading proficiency was not as good as that of the Rank 3 students. This result is explainable from the viewpoint of the different natures of L2 reading proficiency and achievement tests. While proficiency tests involve contents unrelated to the language courses, the contents of achievement tests must be related to course learning in which learners were engaged (Ross, 1998; Bachman and Palmer, 2010). Given that less anxious learners were likely to be more active in L2 classroom learning (e.g., Horwitz et al., 1986; Saito et al., 1999; Horwitz, 2001; Yamashita, 2007; Zhao et al., 2013), it is possible that the Rank 2 students could achieve course learning goals because of relatively low L2 reading anxiety. The weak correlation between L2 reading proficiency and achievement also supports the interpretation that anxiety, self-efficacy, and confidence in L2 reading affected the degree of class engagement and enjoyment more than L2 reading proficiency (Matsuda and Gobel, 2004; Mills et al., 2007). Consistent with Alderson et al. (2016), the student group with low anxiety, but low proficiency can be regarded as being in a developmental stage of L2 reading.

As expected, great care should be taken with Rank 3 students. The results indicated the probability of success in L2 classrooms decreased precipitously when their L2 reading anxiety scores crossed the second threshold of the FLRAS (> = 68). The means of their L2 achievement test were also much lower than Rank 1 and 2 students. Because the majority of students who received a fair or failing grade were classified into Rank 3, the latent rank model has the potential to identify the students being struggling in L2 classrooms. Consistent with Ganschow and Sparks (1991), students who were labeled as potentially unsuccessful in L2 learning were inferior in L2 reading skills. Unlike the students of Rank 2, it is possible that the double bindings caused by low proficiency and high anxiety in L2 reading hurt them, leading to the lowest L2 achievement among the groups. Moreover, the results were consistent with Alamer and Lee (2021) and Sparks and Alamer (2022) that lower L2 achievement increased the magnitude of L2 anxiety. Although the relationships between L2 anxiety and proficiency will determine student achievement in L2 classroom learning (Horwitz, 2001; Dörnyei and Ryan, 2015; MacIntyre, 2017), it is also important to consider that the promising solution to reducing L2 reading anxiety is to develop L2 reading skills.

The present findings emphasize the importance of understanding learners’ affective profiles to classify them into suitable learning environments. Profiling data regarding specific anxieties in response to L2 reading will determine what kind of instruction is necessary for each group. For example, the perceived difficulties in L2 reading differentiated the ranked groups (see Table 4), and the priority should be to improve the skill and knowledge necessary for reading comprehension. This perspective is consistent with the mediation analysis results, in which the participants perceived higher anxiety as a result of lower L2 reading proficiency. After improving the level of L2 reading proficiency, teachers may be able to help the students develop their self-efficacy to reduce L2 reading anxiety further. Given the associational nature of language anxiety and proficiency (Teimouri et al., 2019), the language anxiety scales can be functioned as basic diagnostic testing.

## Conclusion

Most L2 learners perceive L2 anxiety in classrooms, to which teachers do not attribute adequate importance (Tran et al., 2013). Given the clear importance of assessing individual differences in L2 learning, the present study applied the latent rank model to identify struggling students in L2 classrooms. The results showed the FLRAS was not sensitive enough to discriminate L2 reading anxiety on its continuous scale. Instead, the FLRAS could categorize students into three ranked groups according to substantial differences in L2 reading anxiety symptoms. The psychometric function provided by the estimated cutoff points also helped determine success probabilities in L2 classrooms. These findings significantly contribute to improving the learning experiences in L2 classrooms as well as the assessment quality of individual differences in L2 learning.

Toward future research, several factors other than L2 anxiety must be incorporated to identify struggling students in L2 learning. For example, Ganschow and Sparks (1991) showed the predictive power of learners’ L2 learning history, developmental history, academic learning history, and tests and classroom learning characteristics in identifying students with L2 learning disabilities. The present study conducted brief screening in educational settings; therefore, the integration of potential cognitive and affective factors determining L2 achievement will advance theoretical and methodological discussions in research on individual differences in L2 learning.

## Data availability statement

The datasets presented in this study can be found in online repositories. The names of the repository/repositories and accession number(s) can be found at: https://www.iris-database.org/iris/app/home/detail?id=york%3a940393&ref=search.

## Ethics statement

The studies involving human participants were reviewed and approved by Nihon University. The patients/participants provided their written informed consent to participate in this study.

## Author contributions

All authors listed have made a substantial, direct, and intellectual contribution to the work and approved it for publication.

## Funding

This study was supported by Grants-in-Aid for Scientific Research (B) no. 20H01287 and for Young Scientists (B) no. 18K12443 from the Japan Society for the Promotion of Science.

## Conflict of interest

The authors declare that the research was conducted in the absence of any commercial or financial relationships that could be construed as a potential conflict of interest.

## Publisher’s note

All claims expressed in this article are solely those of the authors and do not necessarily represent those of their affiliated organizations, or those of the publisher, the editors and the reviewers. Any product that may be evaluated in this article, or claim that may be made by its manufacturer, is not guaranteed or endorsed by the publisher.

## Footnotes

^1^The standard error of measurement estimates how repeated measures of individuals on the same instrument tend to be distributed around their true score. The formula is *SD**sqrt(1 − Cronbach’s α). Since Cronbach’s α of the FLRAS is generally high (*M* = 0.87), when the *SD* of the FLRAS score is 10, the standard error of measurement will be 3.61 (Teimouri et al., 2019).

^2^Despite a lack of any specific explanation, these cutoff points seem to be determined based on the Likert-scale; for example, the score of 99 indicates that learners are likely to answer “(3) *neither agree nor disagree*” to 33 items.

^3^Based on Sparks et al. (2008), this study recognized students whose grade was fair as being potentially struggling in L2 classrooms because they would have failed the class if they missed a few more points on the achievement test.

^4^Since there were some cases where dependent variables did not satisfy the normality assumption, this study used the non-parametric test to compare the outcomes.

^5^In the latent rank model, the values of item discriminability can be considered in a similar way to factor loadings (Shojima, 2007, 2008, 2009). This study used the conventional.30 and over (Finch and French, 2018) when interpreting the discriminative power of each questionnaire item.

## References

[ref1] Alamer A. Lee J. (2021). Language achievement predicts anxiety and not the other way around: a cross-lagged panel analysis approach. Lang. Teach. Res. doi: 10.1177/13621688211033694 [Epub ahead of print].

[ref2] Alderson J. C. Huhta A. Nieminen L. (2016). Characteristics of weak and strong readers in a foreign language. Mod. Lang. J. 100, 853–879. doi: 10.1111/modl.12367

[ref3] American Educational Research Association (2014). Standards for Educational and Psychological Testing. Washington, D.C.: The Author.

[ref4] Andersen R. W. (1978). An implicational model for second language research. Lang. Learn. 28, 221–282. doi: 10.1111/j.1467-1770.1978.tb00134.x

[ref5] Bachman L. F. Palmer A. S. (2010). Language Assessment in Practice: Developing Language Assessments and Justifying Their Use in the Real World. Oxford, UK: Oxford University Press.

[ref6] Cheng Y.-S. (2017). Development and preliminary validation of four brief measures of L2 language-skill-specific anxiety. System 68, 15–25. doi: 10.1016/j.system.2017.06.009

[ref001] Cheng Y.-S. Horwitz E. K. Schallert D. L. (1999). Language anxiety: Differentiating writing and speaking components. Lang. Learn. 49, 417–446. doi: 10.1111/0023-8333.00095, PMID: 33150970

[ref7] Crowther D. Kim S. Lee J. Lim J. Loewen S. (2021). Methodological synthesis of cluster analysis in second language research. Lang. Learn. 71, 99–130. doi: 10.1111/lang.12428, PMID: 33150970

[ref8] Dörnyei Z. Ryan S. (2015). The Psychology of the Language Learner Revisited. New York, NY: Routledge.

[ref9] Educational Testing Service (2007). TOEIC Bridge® Official Guide & Question Collection. Tokyo, Japan: Institute for International Business Communication

[ref002] Elkhafaifi H. (2005). Listening comprehension and anxiety in the Arabic language classroom. Mod. Lang. J. 89, 206–220. doi: 10.1111/j.1540-4781.2005.00275.x

[ref10] Finch W. H. French B. F. (2018). Educational and Psychological Measurement. New York, NY: Routledge

[ref11] Ganschow L. Sparks R. (1991). A screening instrument for the identification of foreign language learning problems. Foreign Lang. Ann. 24, 383–398. doi: 10.1111/j.1944-9720.1991.tb00484.x

[ref12] Ganschow L. Sparks R. (1996). Anxiety about foreign language learning among high school women. Mod. Lang. J. 80, 199–212. doi: 10.1111/j.1540-4781.1996.tb01160.x

[ref13] Ganschow L. Sparks R. (2001). Learning difficulties and foreign language learning: a review of research and instruction. Lang. Teach. 34, 79–98. doi: 10.1017/S0261444800015895, PMID: 35686064

[ref14] Grabe W. (2009). Reading in a Second Language: Moving From Theory to Practice. New York, NY: Cambridge University Press.

[ref15] Hamada A. Takaki S. (2021a). Approximate replication of Matsuda and Gobel (2004) for psychometric validation of the foreign language reading anxiety scale. Lang. Teach. 54, 535–551. doi: 10.1017/S0261444819000296

[ref16] Hamada A. Takaki S. (2021b). Effects of multidimensional foreign language reading anxiety on achievement in Japanese EFL classrooms. System 101:102613. doi: 10.1016/j.system.2021.102613

[ref17] Hasselblad V. Hedges L. V. (1995). Meta-analysis of screening and diagnostic tests. Psychol. Bull. 117, 167–178. doi: 10.1037/0033-2909.117.1.167, PMID: 7870860

[ref18] Hewitt E. Stephenson J. (2012). Foreign language anxiety and oral exam performance: a replication of Phillips’s MLJ study. Mod. Lang. J. 96, 170–189. doi: 10.1111/j.1540-4781.2011.01174.x

[ref19] Horwitz E. K. Horwitz M. B. Cope J. (1986). Foreign language classroom anxiety. Mod. Lang. J. 70, 125–132. doi: 10.1111/j.1540-4781.1986.tb05256.x

[ref20] Horwitz E. K. (2001). Language anxiety and achievement. Annu. Rev. Appl. Linguist. 21, 112–126. doi: 10.1017/S0267190501000071, PMID: 35815128

[ref21] Jee M. J. (2016). Exploring Korean heritage language learners’ anxiety: ‘we are not afraid of Korean!’. J. Multiling. Multicult. Dev. 37, 56–74. doi: 10.1080/01434632.2015.1029933

[ref22] Lambert C. (2010). A task-based needs analysis: putting principles into practice. Lang. Teach. Res. 14, 99–112. doi: 10.1177/1362168809346520

[ref23] Liu R. Jiang Z. (2018). Diagnostic classification models for ordinal item responses. Front. Psychol. 9:2512. doi: 10.3389/fpsyg.2018.02512, PMID: 30618941 PMC6297886

[ref24] Liu R. Jiang Z. (2020). A general diagnostic classification model for rating scales. Behav. Res. Methods 52, 422–439. doi: 10.3758/s13428-019-01239-9, PMID: 31025254

[ref25] MacIntyre P. D. (1999). “Language anxiety: A review of the research for language teachers,” in Affect in Foreign Language and Second Language Learning. ed. Young D. J. (New York, NY: McGraw-Hill), 24–45.

[ref26] MacIntyre P. D. (2017). “An overview of language anxiety research and trends in its development,” in New Insights Into Language Anxiety: Theory, Research and Educational Implications. eds. Gkonou C. Daubney M. Dewaele J. M. (Bristol, UK: Multilingual Matters).

[ref27] MacIntyre P. D. Gardner R. C. (1991). Methods and results in the study of anxiety and language learning: a review of the literature. Lang. Learn. 41, 85–117. doi: 10.1111/j.1467-1770.1991.tb00677.x

[ref28] Marcos-Llinás M. Garau M. J. (2009). Effects of language anxiety on three proficiency-level courses of Spanish as a foreign language. Foreign Lang. Ann. 42, 94–111. doi: 10.1111/j.1944-9720.2009.01010.x

[ref29] Matsuda S. Gobel P. (2004). Anxiety and predictors of performance in the foreign language classroom. System 32, 21–36. doi: 10.1016/j.system.2003.08.002

[ref30] Mills N. Pajares F. Herron C. (2007). Self-efficacy of college intermediate French students: relation to achievement and motivation. Lang. Learn. 57, 417–442. doi: 10.1111/j.1467-9922.2007.00421.x

[ref31] Oxford R. Ehrman M. (1992). Second language research on individual differences. Annu. Rev. Appl. Linguist. 13, 188–205. doi: 10.1017/S0267190500002464, PMID: 35804430

[ref003] Pae T.-I. (2013). Skill-based L2 anxieties revisited: Their intra-relations and inter-relations with general foreign language anxiety. Appl. Lingust. 34, 232–252. doi: 10.1093/applin/ams041

[ref32] Pastor D. A. Barron K. E. Miller B. J. Davis S. L. (2007). A latent profile analysis of college students’ achievement goal orientation. Contemp. Educ. Psychol. 32, 8–47. doi: 10.1016/j.cedpsych.2006.10.003

[ref33] Phillips E. M. (1992). The effects of language anxiety on students’ oral test performance and attitudes. Mod. Lang. J. 76, 14–26. doi: 10.1111/j.1540-4781.1992.tb02573.x

[ref34] R Core Team (2021). R: A Language and Environment for Statistical Computing. R Foundation for Statistical Computing. Available at: https://www.R-project.org/

[ref35] Ravand H. Baghaei P. (2020). Diagnostic classification models: recent developments, practical issues, and prospects. Int. J. Test. 20, 24–56. doi: 10.1080/15305058.2019.1588278

[ref36] Ross S. (1998). Self-assessment in second language testing: a meta-analysis and analysis of experiential factors. Lang. Test. 15, 1–20. doi: 10.1177/026553229801500101

[ref37] Saito Y. Horwitz E. K. Garza T. J. (1999). Foreign language reading anxiety. Mod. Lang. J. 83, 202–218. doi: 10.1111/0026-7902.00016

[ref38] Sellers V. D. (2000). Anxiety and reading comprehension in Spanish as a foreign language. Foreign Lang. Ann. 33, 512–520. doi: 10.1111/j.1944-9720.2000.tb01995.x

[ref40] Shao K. Yu W. Ji Z. (2013). An exploration of Chinese EFL students' emotional intelligence and foreign language anxiety. Mod. Lang. J. 97, 917–929. doi: 10.1111/j.1540-4781.2013.12042.x

[ref41] Shojima K. (2007). Neural test theory. DNC Research Note, 07-02. Available at: http://shojima.starfree.jp/ntt/Shojima2007RN07-02.pdf

[ref42] Shojima K. (2008). Neural test theory: A latent rank theory for analyzing test data. DNC Research Note, 08-01. Available at: http://shojima.starfree.jp/ntt/Shojima2008RN08-01.pdf

[ref43] Shojima K. (2009). “Neural test theory,” in New Trends in Psychometrics. eds. Shigemasu K. Okada A. Imaizumi T. Hoshino T. (Tokyo, Japan: Universal Academy Press), 417–426.

[ref44] Shojima K. (2019). Exametrika (Version 5.5). [Computer software]. Available at: http://antlers.rd.dnc.ac.jp/~shojima/exmk/index.htm

[ref0001] Sparks R. L. Alamer A. (2022). Long-term impacts of L1 language skills on L2 anxiety: The mediating role of language aptitude and L2 achievement. Lang. Teach. Res. doi: 10.1177/13621688221104392 [Epub ahead of print].

[ref45] Sparks R. L. Humbach N. Javorsky J. (2008). Individual and longitudinal differences among high and low-achieving, LD, and ADHD L2 learners. Learn. Individ. Differ. 18, 29–43. doi: 10.1016/j.lindif.2007.07.003

[ref46] Sparks R. J. Luebbers J. Castañeda M. Patton J. (2018a). High school Spanish students and foreign language reading anxiety: Déjà vu all over again all over again. Mod. Lang. J. 102, 533–556. doi: 10.1111/modl.12504

[ref47] Sparks R. L. Patton J. Luebbers J. (2018b). L2 anxiety and the foreign language reading anxiety scale: listening to the evidence. Foreign Lang. Ann. 51, 738–762. doi: 10.1111/flan.12361

[ref48] Swanson H. L. (2017). A latent transition analysis of English learners with reading disabilities: do measures of cognition add to predictions of late emerging risk status? Top. Lang. Disord. 37, 114–135. doi: 10.1097/TLD.0000000000000117

[ref49] Teimouri Y. Goetze J. Plonsky L. (2019). Second language anxiety and achievement. A meta-analysis. Stud. Second. Lang. Acquis. 41, 363–387. doi: 10.1017/S0272263118000311

[ref50] Tran T. T. T. Baldauf R. B. Jr. Moni K. (2013). Foreign language anxiety: understanding its status and insiders’ awareness and attitudes. TESOL Q. 47, 216–243. doi: 10.1002/tesq.85

[ref51] Wu H. J. (2011). Anxiety and reading comprehension performance in English as a foreign language. Asian EFL J. 13, 273–307.

[ref52] Xiao Y. Wong K. F. (2014). Exploring heritage language anxiety: a study of Chinese heritage language learners. Mod. Lang. J. 98, 589–611. doi: 10.1111/modl.12085

[ref53] Yamashita J. (2007). The relationship of reading attitudes between L1 and L2: an investigation of adult EFL learners in Japan. TESOL Q. 41, 81–105. doi: 10.1002/j.1545-7249.2007.tb00041.x

[ref54] Zhang X. (2019). Foreign language anxiety and foreign language performance: a meta-analysis. Mod. Lang. J. 103, 763–781. doi: 10.1111/modl.12590

[ref55] Zhao A. Guo Y. Dynia J. (2013). Foreign language reading anxiety: Chinese as a foreign language in the United States. Mod. Lang. J. 97, 764–778. doi: 10.1111/j.1540-4781.2013.12032.x

